# 250 labels used to stigmatise people with mental illness

**DOI:** 10.1186/1472-6963-7-97

**Published:** 2007-06-28

**Authors:** Diana Rose, Graham Thornicroft, Vanessa Pinfold, Aliya Kassam

**Affiliations:** 1 Service User Research Enterprise, Health Service and Population Research Department, Institute of Psychiatry, King's College London, London, UK; 2 Section of Community Mental Health Health Service and Population Research Department Institute of Psychiatry, King's College London, London, UK; 3 Rethink severe mental illness, Royal London House, Finsbury Square, London, UK

## Abstract

**Background:**

The stigma against people with mental illness is a major barrier to help-seeking in young people for mental health problems. The objective of this study was to investigate the extent of stigma in relation to treatment avoidance in 14 year-old school students in England in relation to how they refer to people with mental illness.

**Methods:**

This is a qualitative, cross-sectional study. The data were gathered as part of the baseline assessment for an intervention study intended to reduce stigma among 14 year old school students. The participating schools were two grammar (selective) schools and three comprehensive (non-selective) schools. At the start of the lesson, the students were asked 'What sorts of words or phrases might you use to describe someone who experiences mental health problems?' Words and terms used to refer to mental illness were enumerated. Using the grounded theory approach, words and terms were grouped in terms of their denotative and connotative meanings. Labels were then derived to capture the key themes attached by the students to the concepts of mental illness. The frequencies of occurrence for each word were also tabulated.

**Results:**

400 of the 472 participating students (85%) provided 250 words and terms to describe a person with mental illness. Five themes were identified from the data. The first theme called 'popular derogatory terms' (116 items) accounted for nearly half of the words examined. The second theme occurred less often and was described as 'negative emotional state' (61 items). The third theme demonstrated the confusion of young people between physical disabilities, learning difficulties and mental health problems (38 items). The use of psychiatric diagnoses (15 items) and terms related to violence (9 items) were unexpectedly uncommon.

**Conclusion:**

Our findings suggest the hypothesis that help-seeking by mentally ill young people may be improved by interventions that address both their lack of factual information about mental illness, and those which reduce their strong negative emotional reactions towards people with mental illness.

## Background

Most young people who are mentally ill do not seek help [1-5]. Yet mental illnesses among children and adolescents are common, affecting about 10% of young people [6,7]. The rates for some mental disorders, including suicide, are increasing [8,9]. Up to half of those who fail to complete secondary school have mental illness [10]. Those who do, more often turn to friends and family for help than to health professionals [11,12]. Teenagers seek help less often than adults [13]. As few as 4% of young people with a mental illness seek help from a family doctor [14], and consultation rates are especially low among young men [15]. This paper argues that the stigma against mental illness is a powerful (and potentially reversible) contributory factor towards the reluctance of many young people to seek help for mental illness.

Research on help-seeking has paid particular attention to the confidentiality of healthcare, young people's knowledge about services, and how accessible they are [16]. But such factors do not fully explain the very low rates of consultation among young people who are mentally ill [17,18]. Recent work has focussed attention on whether young people know enough to allow them to correctly identify mental illness in themselves or in their peers (so called 'mental health literacy') [19], and upon their emotional/attitudinal responses (and associated stigma) to people with mental illness, as potential explanatory factors for help-seeking or help-avoidance [20].

Stigma is a term which has evaded clear, operational definition [21-24]. It can be considered as an amalgamation of three related problems: a lack of knowledge (ignorance), negative attitudes (prejudice), and excluding or avoiding behaviours (discrimination) [25-27]. In relation to knowledge about mental illness it is clear that there are striking knowledge gaps. [28-30]. For example, in Scotland most children do not know what to do if they have a mental health problem, or what to recommend to a friend with mental health difficulties. Only 1% mentioned school counselling, 1% nominated helplines, 4% recommended talking with friends, 10% said that they would turn to a doctor, but over a third (35%) were unsure where to find help [31].

There is also fairly strong evidence that negative emotions and attitudes act as barriers to care. Compared with adults, young people have less favourable attitudes towards people with mental illness [32]. Conversely, young people with mental illness may be exposed to higher levels of stigma than adults [33]. Commonly young people feel that mental illness is embarrassing [34], should be handled privately, and people with these views tend to seek help less often [35-37]. Attributions for the cause of the condition are also important. Young people who believe that mental illnesses are the responsibility of the person affected are more likely to react to people who are mentally ill with anger, pitilessness or avoidance [38]. There are therefore grounds to consider that stigma may be one important factor in reducing help-seeking for mental illnesses, for example by avoiding the embarrassment of diagnosis [37,39,40].

A recent study investigated whether accurate recognition and labelling of mental disorders by young people (aged 12 to 25 years) is associated with better help-seeking preferences [41]. After being shown a vignette of either a young person with depression or psychosis, each participant was asked what they thought was wrong with the person in the vignette, how long the person should wait to get help and what form of help they should seek. The results showed that the young people who correctly labelled the disorder were also those who most identified appropriate help-seeking and treatment options. Although the Wright et al. study explored help-seeking directly, the stigmatising attitudes and beliefs held by young people towards mental illness and people with mental illness which may deter them from seeking help were not explored. The purpose of our study was to determine what young people actually think about mental illness/people with mental illness and explore the type of language they use to label it.

## Methods

Much stigma-related research has used vignettes or social distance scales which may constrain what respondents can express about stigma. Our method was intended to allow young people to express what they thought about mental illness in a way that was not pre structured by attitude scales or vignettes. To explore the role of stigma in relation to treatment avoidance further, we describe here the terms used by 14 year-old school students in England to refer to people with mental illness. These data were gathered as a part of the baseline assessment in an intervention study intended to reduce stigma among school students. Full details of the method are given elsewhere [42]. Briefly, members of Mid-Kent Mental Health Awareness group, including service users, delivered two educational sessions in 5 local secondary schools. Two educational lessons, each one hour long, were given within the Personal Social Health and Education curriculum students aged 14. The participating centres were two grammar (single-sex selective state) school, and three comprehensive (co-educational, non-selective state) schools, typical of those in the local area. At the start of the lesson the young people filled out baseline questionnaires where they were asked 'What sorts of words or phrases might you use to describe someone who experiences mental health problems?' The project was approved by the local research ethics committee.

The data analysis was deliberately straightforward. First we enumerated the words and terms used to refer to mental illness. Although some of the young people elaborated a little upon the words they chose, most of the data consisted of single words. The first part of the analysis was to tabulate them in order of frequency where the words or terms were offered by at least 3 different students. This was done to map the meanings that students gave to mental illness in terms of their relative importance. Secondly, using the approach of grounded theory [43], the words were grouped in terms of both their denotative and connotative meanings, and labels were derived which captured the key themes attached by the young people to these concepts of mental illness. Denotative meanings are what a term refers to, what it 'names', and connotative meanings are the associations, values, and judgements that surround this. A preliminary examination of our material suggested that connotative meanings would be very significant. We went on to calculate the frequencies of occurrence for each category, and finally over-arching concepts were derived.

## Results

Of the 634 14-year old students identified in the four schools, 472 (74%) students received both of the two mental health awareness workshops and completed baseline and follow-up assessments. Of these 400 (85%) pupils provided 250 words and terms, and 20 longer phrases, to describe a person with a mental health problem in their baseline assessment. The sample was predominantly female (73%). Fifty two per-cent of the sample attended co-education state schools whilst 48% attended single sex grammar schools. Two hundred and eight students (52%) reported that they personally knew someone with mental illness.

Table 1 show the 44 most frequently occurring words and terms, namely those that were stated by 3 or more students. Three quarters (n = 33) of these terms are strongly negative in referring to people with mental health problems. Seven terms (16%) of those shown were broadly neutral, including the use of medical diagnostic terms, and only 4 (9%) could be described as at all empathic or eliciting compassion, 'sad' or 'isolated'.

**Table 1:** Most frequently occurring words and terms

**Term**	**Frequency**	**Term**	**Frequency**
Disturbed	11	Scary	5
Nuts	11	Div	4
Confused	10	Dumb	4
Psycho	10	Ill	4
Spastic	10	Loneliness	4
Crazy	9	Loony bin	4
Depression	7	Psychiatric	4
Disabled	7	Screw loose	4
Mad	7	Stress	4
Unpredictable	7	Violence	4
Insane	6	Brain dead	3
Loony	6	Demanding	3
Mental	6	Demented	3
Schizophrenia	6	Dinlo	3
Thicko	6	Distressed	3
Weird	6	Embarrassed	3
Depressed	5	Flid	3
Different	5	Frustrated	3
Freak	5	Isolated	3
Odd	5	Sad	3
Problem	5	Strait jacket	3
Retard	5	Wheel chairs	3

We identified five themes which emerged from these data (Table 2). The labels used for these themes reflect the overwhelmingly negative connotations used by young people to describe people with mental health problems. The first theme accounts for nearly half the words (116 items) we examined. We have termed this 'popular derogatory terms', and they are in effect 'slang'. In terms of the distinction between denotative and connotative meaning, these terms appear to have no referent but are a set of negative associations and judgements in and of themselves. The second theme occurred about half as often as the first, and is described as 'negative emotional state' (61 items). Not one positive emotional state was mentioned. The most frequently mentioned words were 'disturbed' and 'confused'. These are powerful terms and appear to reflect anxiety on the part of respondents when thinking about mental health problems and the people affected by them.

**Table 2:** Super-ordinate categories emerging from the terms used

**Theme**	**1 Popular derogatory terms**	**2 Negative Emotional State**	**3 Physical Illness or Learning Disability**	**4 Psychiatric categories**	**5 Violence**	**6 Loneliness**
Number of instances	114	61	38	15	9	10
Examples	Nuts	Disturbed	Disabled	Schizophrenia	Scary	Isolated
	Psycho	Confused	Spastic	Depression	Violence	Loneliness
	Crazy	Depression	Dumb	Psychiatric		
	Loony	Ill	Demented			
	Weird	Stress	Wheelchairs			
	Freak	Distressed				
	Screw Loose	Embarrassed				

The third theme demonstrates confusion by the young people between physical disabilities, learning difficulties and mental health problems (38 items). It is notable that the young people hardly used formal psychiatric diagnoses (the fourth theme) at all, preferring the use of emotionally-charged negative terms which represent people with mental illness as someone having a physical disability (15 items).

Against our expectations, the fifth theme of violence was relatively rare (9 items). This is surprising given that psychiatric patients are so often portrayed by the UK media as perpetrators of violence. We have no explanation for why the theme of violence was used in such a limited way, except to say that many of the derogatory terms have a covert connotation, referring to something to be feared. Only two terms made up the final theme of sadness and isolation, but they do have a slightly more positive connotation than the rest of the material. Isolation and loneliness suggest pity rather than fear.

More striking was the sheer range and emotional power of the words used (n = 250) showing both a remarkable virtuosity (encroaching upon the vulgar) and a lack of precision in how students expressed themselves when speaking about people with mental illness (see Table 3).

**Table 3:** Terms used by 14 year old school students to refer to mental illness

Abnormal	Frustration	Not fair	Sometimes lacking brain power
Abusive	Fucked	Not happy	Spakka
Alone	Funny	Not obvious	Spanner
Alzheimers	Gay	Not quite there	Spastic
Angry	Get lost	Not the sharpest knife in the drawer	Spaz
Anti-social	Gone in the head	Numscull	Split personality
Asylums	Goon	Nutcase	Spoone
Attention seekers	Green room	Nutter	Stiggy nutter
Autism	Halfwit	Nuts	Stigma
Bewildered	Hallucinating	Nutty as a fruitcake	Strait jackets
Bimbo	Hallucinations	OCD	Strange
Bonkers	Hand fed	Odd	Stress
Brain damage	Handicapped	Oddball	Stressed
Brain dead	Happy club	Off their rocker	Therapist
Breakdown	Hard	Out of it	Therapy
Childish	Hard work	Outcast	Thick
Cola sweat	Head banging	Padded cells	Thicko
Confused	Head case	Paedophile	Thicky
Crackers	Helpless	Panicked	Tiring
Crazy	Hurting yourself	Paranoid	Too much pressure
Cushioned walks	Idiot	Patch Adams	Touchy to talk to
Dangerous	Ill	People who are obsessed	Troubled
Deformed	Indecisive	Perfectly normal	Twisted
Demanding	Infixed in bad habits	Perverted	Twister
Demented	Insane	Physical problems	Ugly
Depressed	Insecure	Physically ill	Unable to make decisions
Depression	Intellectually challenged	Pills	Unappreciated
Deranged	Intimidating	Pinflump	Unapproachable
Difficulty learning	Irrational	Pive	Uncomfortable
Dildo	Isolated	Plank	Under pressure
Dinlo	Joe from Eastenders	Ponce	Understandable
Disabled	Jumpy	Pressure	Unfair
Disarmed	Learning difficulties	Pressurising families	Unfortunate
Disorientated	Lonely	Problems	Unhappy
Distorted	Loony	Psychiatric	Unpredictable
Distressed	Loony bin	Psychiatric health	Unstable
Distressing	Loser	Psychiatrist	Upsetting
Disturbed	Lost	Psycho	Veg
Disturbing	Lunatic	Psychopath	Vegetable
Disturbing images	Mad	Reject	Victim
Div	Made fun of	Retard	Victimised
Dizzy	Madness	Sad	Violence
Doctors	Manic depression	Sandwich/pepperoni short of a picnic	Violent
Dofuss	Mass murderers	Scared	Voices
Dopy	M.E.	Scared to talk to if they were a murderer or rapist	Voices in your head
Downy	Mental	Scary	Vulnerable
Dribbling	Mental hospital	Schizo	Wacky
Drugged-up	Mental illness	Schizophrenia	Wally
Dulally	Mental institution	Schizophrenic	War
Dumb	Mentally challenged	School can cause it	Wheelchair jockey
Embarrassed	Mentally handicapped	School pressure	Weird
Embarrassing	Mentally ill	Screw loose	Weirdo
Empty	Misunderstood	Screwed	Wheel chairs
Escaped from an asylum	Mong	Sees things in a different way	White coats
Excluded	More common than you think	Segregation	Wild
Feel sorry	Muppets	Self-harm	Wild funny noises
Few sandwiches short of a picnic basket	Needing help	Shock syndrome	Window licker
Flid	Nervous	Shouts	Withdrawn
Flip in the head	Nightmares	Sick in the head	World of their own
Freak	Non-caring	Simple	Worried
Fruit cake	None caring	Simpleton	You belong in a home
Frustrated	No-one upstairs	Some people born mentally ill	
Frustrating	Not all there	Sometimes includes drugs	

## Discussion

How do young people learn such wide-ranging, emotionally-charged and negative terms about mental illness? The primary sources appear to be from the media, and from family and peers [44-46]. Derogatory references about people with mental illness appear commonly in the print, broadcast and cinematographic media [47,48]. For television and newspaper items about mental illness, for example, between one third and two thirds refer primarily to violence [25]. The highest rate of such negative coverage occurs in children's animations, where up to two-thirds of all references are to violence [49,50]. Interestingly, almost a half (46%) of all the episodes contained some reference to mental illness, especially in cartoons, where the vocabulary analysed in one New Zealand study was '*predominantly negative fundamentally disrespectful. The characters were typically losing control, constantly engaged in illogical and irrational actions'*, and were *'stereotypically and blatantly negative, and served as objects of amusement, derision or fear*.' [50] Children's programmes in the USA have produced almost identical results, where the images were '*typically used to disparage and ridicule*' [44,51]. More specifically, a Canadian study examined Disney animated films for children and found that 85% contained verbal references to mental illness and they were mainly used to '*set apart and denigrate' *the characters [52].

Our results, alongside previous research, suggest the following conclusions. First, the level of factual knowledge among 14 year old school children about mental illness is remarkably low, and this may partially explain why their rates of recognition of mental illness are poor. The magnitude of this information gap has previously been underestimated [53]. Second, the strongly negative emotions described in this paper offer a route for future investigation on whether this helps to explain why young people, even more than adults [54-56], are so reluctant to seek help when experiencing mental illness, and often tend to feel that they should cope alone [57].

Our methodological approach has three important limitations. First, our method of data collection may be described as over-simplistic. However, our method has given a clear account of the full range of language used by young people when referring to mental illness which would be difficult otherwise to ascertain and so this study can be used as a benchmark for future research. Second, as the study was predominantly female, the sample size did not allow us to explore important possible gender differences, for example whether the words and terms used suggested a greater degree of mental health literacy for female students [58]. Third, the nature of the results, very largely showing the use of negative terminology, did not allow us to establish whether those students with personal contact with people with mental illness used systematically more favourable terms.

## Conclusion

An appreciation of both factual ignorance and the degree of emotionally-charged prejudice by school students against people with mental illness is necessary when planning interventions intended to improve help-seeking [25,59,60]. The strongest evidence-based intervention known to reduce stigmatising attitudes (but not yet shown to change discriminatory behaviour) is direct social contact with a person who has mental illness [21,26,42,61,62]. Our findings, if replicated, suggest that help-seeking by mentally ill young people may be improved by interventions that address both their lack of factual information about mental illness, and those which reduce their strong negative emotional reactions towards people with mental illness [63].

## Competing interests

The author(s) declare that they have no competing interests.

## Authors' contributions

GT and VP secured the grant for this work, and conducted the study. DR, AK, GT and VP together analysed and interpreted the data for the part of the study reported in this paper. All authors read and approved the final manuscript.

## Pre-publication history

The pre-publication history for this paper can be accessed here:


